# Exploring the Personal Recovery Construct in Bipolar Disorders: Definition, Usage and Measurement. A Systematic Review

**DOI:** 10.3389/fpsyt.2022.876761

**Published:** 2022-06-23

**Authors:** Marion Chirio-Espitalier, Benoit Schreck, Melanie Duval, Jean-Benoit Hardouin, Leila Moret, Marie Grall Bronnec

**Affiliations:** ^1^Nantes University, CHU Nantes, UIC Psychiatrie et Santé Mentale, Nantes, France; ^2^Nantes University, Univ Tours, CHU Nantes, INSERM, MethodS in Patients Centered Outcomes and HEalth ResEarch, SPHERE, Nantes, France; ^3^Department of Public Health, University Hospital of Nantes, Nantes, France

**Keywords:** bipolar disorder, personal recovery, mental health recovery, patient-reported outcome measures, recovery-oriented practice, systematic review

## Abstract

Personal recovery from psychiatric disorders is a journey toward a satisfying and hopeful life despite the possible persistence of symptoms. This concept has gained interest and become an increasingly important goal in mental health care programmes. Personal Recovery is well described in the context of severe mental illnesses in general, but little is known about this journey in bipolar disorders and the factors underlying it. A systematic review was conducted according to the PRISMA recommendations, focusing on studies exploring personal recovery in bipolar disorder specifically. The latter have integrated a comprehensive approach to the concept, the existing means of measurement or have explored the levers of recovery in care. Twenty-four articles were selected, including seven qualitative, 12 observational, and five interventional studies. The Bipolar Recovery Questionnaire was the only scale developed *de novo* from qualitative work with bipolar people. Personal recovery did not correlate very closely with symptomatology. Some elements of personal recovery in bipolar disorder were similar to those in other severe mental illnesses: meaning in life, self-determination, hope, and low self-stigma. Specific levers differed: mental relationships with mood swings, including acceptance and decrease in hypervigilance, and openness to others, including trust and closeness. The studies highlighted the role of caregiver posture and the quality of communication within care, as well as the knowledge gained from peers. The choice to exclude articles not focused on bipolar disorder resulted in the provision of very specific information, and the small number of articles to date may limit the scope of the evidence. New components of personal recovery in bipolar disorder emerged from this review; these components could be taken into account in the construction of care tools, as well as in the caregiving posture. Strengthening skills of openness to others could also be a central target of recovery-focused care.

## Introduction

Traditional psychiatry usually considers recovery to be a clinical or functional outcome. Gradually, the concept of “personal recovery” (PR) has emerged and gained interest in the field of psychiatric disorders. PR is distinct from clinical and functional recovery: it refers to a psychological process of adjustment to a disorder rather than to a reduction in symptoms (clinical recovery) or functional improvement (functional recovery). These concepts of clinical recovery, functional recovery and PR are distinct but related (1). This construct was first developed by patients themselves (2, 3) through first-person accounts. The possibility of a favorable outcome of severe psychiatric illnesses, despite the possible persistence of certain symptoms, subsequently aroused the interest of clinicians and public authorities. Indeed, it was found that patients with severe mental illnesses (SMIs), such as bipolar disorder (BD), expressed dissatisfaction with the current primary targets of treatment; they wanted caregivers to place more emphasis on PR outcomes (3).

Anthony et al. proposed the following definition of PR in 1993: “*a deeply personal, unique process of changing one's attitudes, values, feelings, goals, skills and/or roles… a way of living a satisfying, hopeful and contributing life even with the limitations caused by illness*” (4). Through a literature review in 2011, Leamy et al. created the “CHIME framework”, a conceptual framework based on five processes that were identified as important factors of PR: connectedness, hope and optimism about the future, identity, meaning in life, and empowerment in severe mental health problems (5).

PR- and recovery-oriented practices have become important in mental health care and are developing based on the initiative of public authorities and at the request of users and families. A review of PR in BD was carried out by Jagfeld et al. in 2021 (6) and focused on qualitative studies. It showed a greater importance of self-management of the disease and medication, as well as socially significant roles such as work and parenthood, and brought a notion of tension inherent in the RA process. This tension would require an active process of acceptance.

A recent scoping review of systematic reviews in PR identified two needs: to better understand the process of PR, in particular the underlying mechanisms of PR, and to adapt the CHIME conceptual framework to the characteristics of specific populations, in particular those with mood disorders (7). This conceptual framework has been studied in the context of schizophrenia or SMIs in general; nevertheless, little is known about factors contributing specifically to PR in people with BD.

It is important to pay attention to psychological and environmental factors underlying PR in BD specifically. Indeed, it is likely that some elements of PR in BD are found in SMIs in general, while others may be more specific to BD. The mood fluctuations constitutive of BD and the relative need for balance may alter the constituents and determinants of PR; for example, high levels of optimism, involvement in meaningful activities, social interaction and self-confidence may be related to an imbalance in the disorder, which may not be the case for people with psychosis (8).

The present systematic review sought to address the following question: What is the current evidence regarding the process of PR in people with BD?

## Methods

### Search Strategy

A systematic review was conducted to identify all relevant publications using PsychINFO, MEDLINE, ScienceDirect. We complied with the Preferred Reporting Items for Systematic reviews and Meta-Analyses (PRISMA) (9). The search terms used were “bipolar disorder“ associated with “personal recovery” or with “mental health recovery”. Indeed, both of the latter two terms were found to describe the process of PR in published studies. These terms needed to appear in title, abstract or keywords. Duplicates were eliminated.

**Figure 1:**
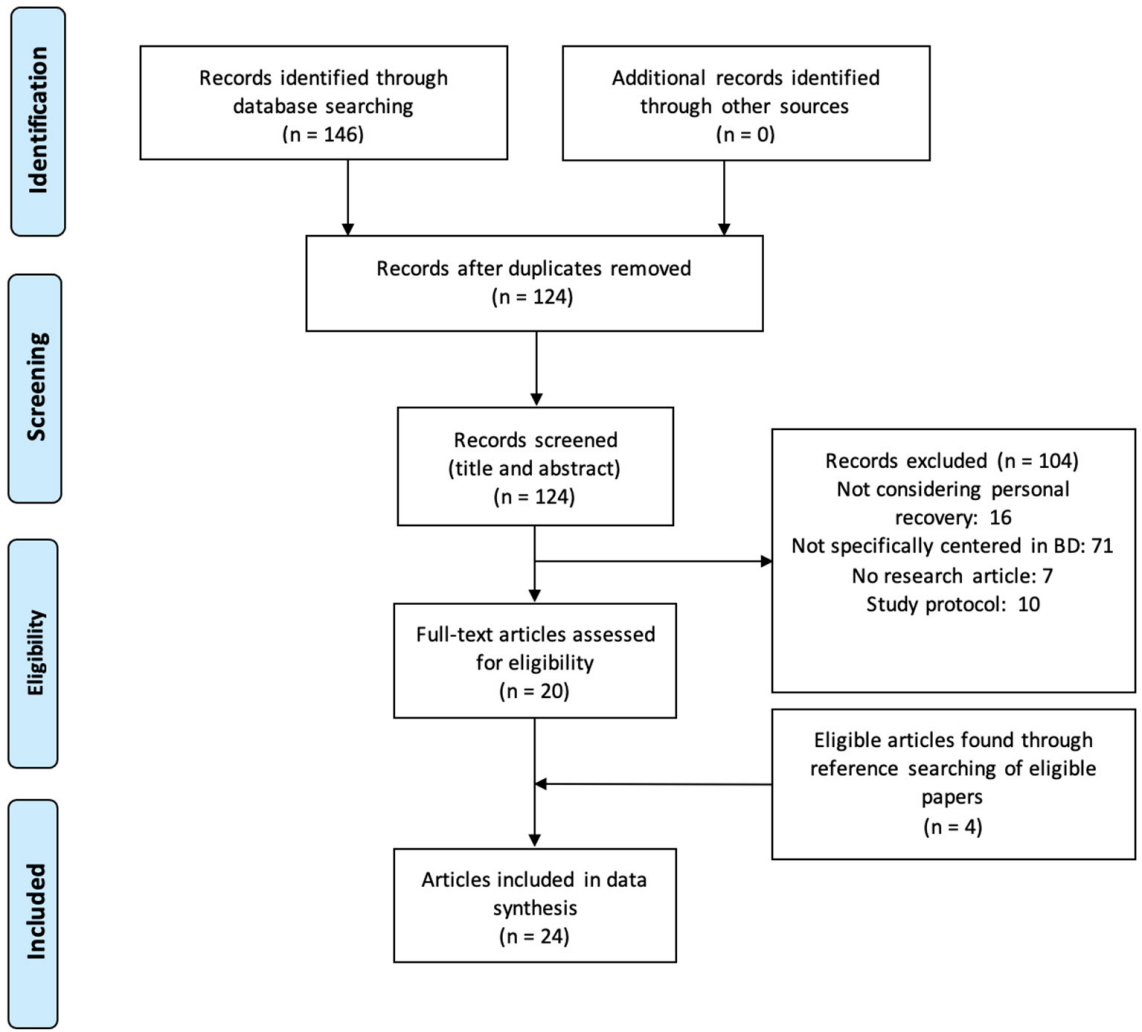
Flowchart.

Figure 1 presents the flowchart illustrating the search and identification of relevant articles.

### Eligibility Criteria

Relevant articles were selected based on the following inclusion criteria: peer-reviewed studies available in English or French with the full text available that were published prior to 1 May 2021 and that focused on PR in people suffering from BD. No backward time constraints were used. Three types of studies were selected: qualitative studies, interventional studies, and observational studies.

Articles that met the following exclusion criteria were excluded: first-person accounts and case reports, study protocols that had no results yet available, studies of multiple psychiatric disorders, and studies not specifically focusing on PR.

### Article Selection

First, articles were selected based on their titles and abstracts. Second, the full texts of all of the included articles were read. The authors (MCE and MD) performed this work independently and blindly using the same bibliographic search criteria. In the event of disagreement, the relevant articles were discussed with a third author (BS). Additional publications were found by reviewing the citations of the included papers.

### Data Extraction

The articles were classified into three types: qualitative, interventional and observational studies.

Data extraction was differentiated according to the type of article's pre-established grids.

For all studies, data were extracted from the articles and reported by data type as well as country of the research group, type of study, sample size, scale assessing PR/description of PR, correlated dimensions or outcomes, and significant findings/main themes.

### Data Analysis and Synthesis

Thematic analysis was conducted through a line-by-line analysis of included papers coding relevant text. The analysis of the data was carried out using a mainly inductive approach, without a pre-established thematic grid.

The identified articles were analyzed using a thematic analysis that incorporated the following themes: identification of how PR was measured in the various works, a comprehensive approach to PR in BD, and how care may contribute to PR. Through these three themes, we presented the results of different types of work: qualitative studies that described this process, as well as a number of observational and interventional studies exploring the measurement of PR and the factors involved in this process. Additionally, we presented the various interventional studies that targeted PR as an outcome to better understand the evidence for care tools that might contribute to it.

For the content analysis of the qualitative studies, themes and subthemes were extracted from all studies.

The level of scientific evidence of the published studies was classified according to a decreasing level of scientific evidence, in accordance with the guide for the analysis of the literature and the gradation of recommendations, published by the Haute Autorité en Santé (HAS) in 2013 (10). This grid was adapted from the Sackett score (11).

## Results

### Study Characteristics

Twenty-four articles met the inclusion criteria (flowchart in Figure 1). The study characteristics and main results are presented in tables according to the types of studies: qualitative studies (Table 1), observational studies (Table 2), and interventional studies (Table 3).

**Table 1:** Characteristics and main results of qualitative studies included (*N* = 7).

**Research group**	**Country**	**Intervention/ approach**	**Methodology**	**Sample size**	**Findings**
Mansel et al. (12)	UK	Individual in-depth interview	Qualitative study	*N =* 11	Differences between:Ambivalent approaches (avoiding mania, taking medication, identity following diagnosis) Helpful approaches (understanding, life-style fundamentals, social support, and social change)
Todd et al. (13)	UK	Focus Groups	Qualitative study	*N =* 12	PR is not about being symptom free PR requires taking responsibility for your own wellness Self-management is a key component Overcome barriers to recovery: negativity, stigma and taboo
Veseth et al. (14)	Norway	Individual in-depth interview	Qualitative study	*N =* 13	Handling ambivalence about letting go of manic states; Finding something to hang on to when the world is spinning around; Becoming aware of signals from self and others; Finding ways of caring for oneself.
Maassen et al. (15)	Netherlands	Focus groups	Qualitative study	*N =* 56 (7FG)	To formulate the care needs for people with BD Need help for acceptance and find self-care strategies
Crowe et al. (16)	New Zealand	Individual in-depth interview	Qualitative study	*N =* 30	What was helpful in psychotherapy? What do they use 5 years later for their own recovery? Facilitate self-awareness and understanding of BD. Develop self-care strategies, and sense of agency. Emphasize hope and optimism
Tse et al. (17)	China	In-depth interviews	Qualitative study	*N =* 32	How to share Knowledge? The importance of how knowledge has been shared rather than the type of knowledge (technical vs expert-by-experience). – Empathy – Hope-instilling manner – Role models
Retzer et al. (18)	UK	In-depth interviews, Focus groups and modified Delphi process	Qualitative study	*N =* 50 (Outcomes); *N =* 14 (Delphi process)	Construction of a COS (core outcome set) of 11 outcomes

**Table 2:** Characteristics and main results of observational studies.

**Research group**	**Country**	**Intervention/ approach**	**Methodology**	**Sample size**	**Scales assessing PR**	**Other outcomes**	**Findings**	**Quality appraisal of studies (Level I-IV)**
Jones et al. (8)	UK	Online survey	Cross sectional online study + Test-retest 1 month	*N =* 60	BRQ	Symptoms: MRS, HDRS, BDI-II, ISS Functioning: PSP, MOS SF12 Post Traumatic Growth Inventory: PTGI	Validation study of the BRQ scale: reliability, internal consistency, validity. Correlations: Functioning and wellbeing more than symptoms. Post-traumatic growth. Coping and confidence in one's resources	IV
Tse et al. (19)	China	Clinical Interviews	Cross sectional study	*N =* 75	SRS	Recovery-Elements Assessment Questionnaire-patient version (REAQ) Organizational Climate Subscale (OCS) Symptoms (HAM-D- YMRS)	Correlations with latter stages of PR: ≪ Respect, hope, and self-directed empowerment ≫ dimension of REAQ, ≪ meaningful role ≫ dimension Older age Early first diagnosis, Binge drinking history	IV
Tse et al. (20)	China	Clinical Interviews	Cross sectional study	*N =* 75	SRS	Residential status employment status (i.e. functional recovery) Symptoms (HAM-D- YMRS) Elements Assessment Questionnaire-patient version (REAQ)	Correlations with latter stages of PR: Female, being married, Functional recovery and personal recovery are correlated (rs.21–0.28), but far from identical. No significant correlations be-tween SRS scores, residential status, and employment status.	IV
Grover et al. (21)	India		Cross-sectional study	*N =* 185	RAS	Internalized Stigma of Mental Illness Scale (ISMIS), Brief Religious coping scale (RCOPE), Duke University Religiosity Index (DUREL), Religiousness Measures Scale (RMS), Symptoms (HDRS, YMRS) Functioning (GAF)	Correlations: Negative correlation with residual depressive symptoms Positive with: Low internalized stigma High level of functioning Higher use of positive religious coping mechanisms Employment status and high income. No correlation with sociodemographic variables	IV
Dodd et al. (22)	UK	Online survey	Cross sectional online study	*N =* 87	BRQ	Mood symptoms Appraisals and beliefs about mood swings	Correlations PR with: Symptoms: negative correlations with current depression, positive with recent depression, no correlation with mania.	IV
						Positive beliefs about mood swings correlated with High PR/ negative illness models linked to poor PR No correlation with: age, gender, years since diagnosis, medication use, educational level Positive correlation between PR and being in work.	
Etchezarraga et al. (23)	Spain	Online survey; T1-T2 at 6 months	Cross-sectional and longitudinal online study	*N =* 125 (baseline); *N =* 60 (6 months)	BRQ	Resilience Questionnaire for Bipolar Disorder [RBD] Symptoms (ISS) Functioning (Work and Social Adjustment Scale) Quality of Life (Brief-QoL BD)	Correlations PR with: Self-care, self-management and self-confidence (resilience factors of the RBD scale) Self-confidence directly predicted the increase of PR over time. Interpersonal support and self-care indirectly predicted the increase of personal recovery through the mediation of improved self-confidence.	IV
Kraiss et al. (24)	Netherlands	Online survey	Cross sectional online study	*N =* 102	QPR (Questionnaire about the Process of Recovery) 15 items	Well-being (Mental Health Continuum MHC-SF) Short Social Role ParticipationQuestionnaire (S-SRPQ) Symptoms (HADS, ASRM)	Validation study of the QPR scale: reliability, internal consistency, validity. Unidimensional structure. Correlations PR with: Strong positive correlation with wellbeing Positive correlation with social role participation Only weak correlation with manic symptoms.	IV
Kraiss et al. (25)	Netherlands	Online survey	Cross sectional online study	*N =* 107	QPR (Questionnaire about the Process of Recovery) 15 items	Responses to Positive Affect (RPA) Well-being (14-item Mental Health Continuum MHC-SF) Social role participation (S-SRPQ) Symptoms (HADS, ASRM)	Validation study of the QPR scale in which QPR is involved Correlations PR with: Negative correlation with dampening Positive correlation with scores of emotion-focused positive ruminations	IV
Dunne et al. (26)	Australia	Online survey	Cross-sectional study	*N =* 312	BRQ	Self-reported depression or mania	Correlations PR with: Positive correlation with: Quality of Social support Being employed Higher education Negative correlation with: Depressive or manic symptoms.	IV
Mezes et al. (27)	UK	Online survey; T1-T2 at 6 months	Cross-sectional and longitudinal online study	*N =* 107 (baseline); *N =* 90 (6 months)	BRQ	Number of episodes (SCID) Symptoms (CES-D, AMRS) Cognitive Vulnerability Model. (IDQ, HIQ, DAS-24, Response style theory Response Style Questionnaire (RSQ) revised version Behavioral Activation System (BAS) Dysregulation Model. Impulsivity (BIMP)	Correlations PR with: Being in employment/meaningfully occupied predict improved PR at a six-month follow-up Cognitive vulnerability model: negative self-dispositional appraisals are associated with lower PR; high adaptative coping and risk-taking correlated with higher PR.	IV
Wynter et al. (28)	Australia	Online survey	Cross-sectional study	*N =* 393	BRQ	Parental and intimate relationship functioning: Social Adjustment Scale Self-Report (SAS-SR)	Correlations PR with: Being employed Level of education Greater parental functioning and intimate functioning No correlation with: child at home, or living with an intimate partner	IV
Kraiss et al. (29)	Netherlands	Online survey	Cross-sectional study	*N =* 209	QPR	Social Role Participation (S-SRPQ) Symptom anxiety-depression (HADS-A), manic ASRM. Positive emotion regulation (RPA)	Correlations PR with: Satisfaction with social roles = the strongest correlate of personal recovery Dampening: small negative correlation Emotion-focused Positive ruminations: positive correlation Negative correlation with anxiety symptoms; positive correlation with manic symptoms	IV

*N = 12*.

**Table 3:** Characteristics and main results of interventional studies (*N* = 5).

**Research group**	**Country**	**Intervention/ approach**	**Methodology**	**Sample size**	**Scales assessing PR**	**Other outcomes**	**Findings**	**Quality appraisal of studies (Level I-IV)**
Todd et al. (30)	UK	Web-based self-management intervention ≪ living with bipolar ≫ LWB	RCT	*N =* 122 LWB+TAU vs.TAU TAU only (waiting list)	BRQ	Quality of Life (QoL) Qol-BD brief WhoQoL bref Internal States Scale (ISS) The Social Adaptation Self-Evaluation Scale (SASS)	The most robust potential treatment effects QoL, recovery and wellbeing The most important effect size (0,7) for Recovery. The existence of an online community appears to play a key role.	II
Jones et al. (31)	UK	Recovery-focused CBT for recent-onset BD	RCT with 15 months follow-up	*N =* 67 BD recent onset (past 5 years) Therapy vs TAU	BRQ	Time to relapse Clinical symptoms QoL Social functioning Medication adherence Therapeutic alliance	Greater improvement in recovery after therapy, sustained at follow-up (P = 0,010) Time to recurrence was statistically significant for mania and depression (P <0.006) No impact on mood or treatment adherence, Nonsignificant trend toward a positive effect on quality of life and social functioning.	II
Jones et al. (32)	UK	10-session group psychoeducation intervention (Mood on Track) MOT	Pre-post therapy	*N =* 202	BRQ	QoL BD, ISS Anxiety, depression, Work and Social Adjustment Scale WASAS	Recovery. BRQ scores improved between pre and post therapy, difference was of medium effect size and statistically significant. QoL and social functioning improved significantly	IV
Richardson et al. (33)	UK	12 weeks group	Pre-post therapy	*N =* 23	BRQ	Self-esteem and stigma: Views on Manic Depression Questionnaire The Brief Illness Perception Questionnaire	Recovery: significant change in scores on the BRQ p <0.05 Significant changes on the Brief Illness Perception questionnaire post- group	IV
Enrique et al. (34)	Ireland	Internet-delivered self-management intervention for 10 weeks +TAU “Bipolar Toolkit ”	Pre-post therapy	BD; *N =* 20	BRQ	QoL BD Brief Illness Perception Questionnaire (BIPQ) The Internal State Scale [ISS, (35)]	Significant differences for the BRQ (z = 2.38, p =0.017).	IV

Even though no backward time constraints were used in the selection of articles, no articles published prior to 2010 met the selection criteria with these keywords.

#### Study Types

Among the 24 articles selected, we found seven qualitative studies, including one on a core outcome set (COS); 12 observational studies; and five interventional studies, including two randomized controlled trials (RCTs).

Qualitative studies consisted of the systematic analysis of the verbatim narratives of people suffering from BD, collected during individual interviews or focus groups; this analysis allowed us to group the elements of the discourse into themes and subthemes to draw out their meaning. The techniques used were in-depth individual interviews (12, 14, 16, 17) or focus groups (13, 15). The number of subjects included varied from 11 (12) to 56 (15).

Observational studies involved different data collected through objective clinical or sociodemographic indicators and/or subjective psychometric scales at a defined time. Among the 12 observational studies collected, three were validation studies of psychometric scales (8, 24, 25).

Five interventional studies were included in this review. Only two articles reported on RCTs, whereas three reported on pre-post therapy uncontrolled trials.

#### Quality Appraisal of Studies Included

The observational and interventional studies had a rather low level of scientific evidence (level 4), with the exception of the two RCTs (level 2).

### How Is PR Measured in BD?

In this review, we were interested in the measurement tools for PR used with people with BD. In total, we identified four scales assessing PR.

Only two of these PR scales were specifically validated for BD: the Bipolar Recovery Questionnaire (BRQ) (8) and the Questionnaire of Personal Recovery (QPR) (24).

The BRQ was the only scale that has been developed *de novo* from qualitative work among people suffering from BD (13). Despite a moderate sample size (60 subjects), the scale showed good internal consistency (alpha = 0.875). External construct validity was established by incremental differences in personal growth, functioning, mood symptoms and wellbeing. The validation study of the BRQ (8) showed good test-retest reliability at 1 month (r = 0.866, *p* < 0.001). The sample size did not permit the exploration of its factor structure and dimensions.

The QPR was developed from the CHIME framework. The scale was initially created and validated for PR in schizophrenia (36). It was then reduced from 22 to 15 items for a more robust version. Its validation for BD was later performed by Kraiss et al. in 2019 through a cross-sectional survey that included 102 people: the scale showed good internal consistency on a unidimensional scale (α = 0.92). Convergent validation measures assessed wellbeing, social role participation, and symptomatology (24).

Other psychometric scales, nonspecific to BD, were used in the studies. The Recovery Assessment Scale (RAS) was chosen by Grover et al. (21) in a psychometric study. The factor structure of the RAS scale was explored by Corrigan et al. (37) in a population of people suffering from SMIs. This scale had previously been used in an Australian study (38) with a population of severely mentally ill people, of whom only 38 were affected by BD. The small sample of BD patients did not allow reliable conclusions to be drawn in this first work. Similarly, the Chinese team of Tse et al. (19, 20) used the Stages of Recovery Scale (SRS), a scale previously developed with a population of people with SMIs in Taiwan. In these two studies, Tse et al. used the SRS in a sample of 75 people suffering from BD, in which Cronbach's alpha for the SRS was 0.95.

At the date of this review, all interventional studies, whether controlled or uncontrolled, have used the BRQ scale. As a result, the BRQ scale has the most evidence in terms of sensitivity to change.

Characteristics of psychometric scales assessing PR in BD are presented in Table 4.

**Table 4:** Characteristics of scales assessing PR in BD.

**Scale**	**Number of items**	**Internal consistency**	**Dimensions**	**Specific to BD?**	**Sample size of validation study**	**Particularities of the scale**
BRQ	36	Good (α= 0.875) + Good test-retest reliability at 1 month (r = 0.866, *p* <0.001)	Not documented	YES	*N =* 60	Specifically constructed for people with BD
QPR	22 items, secondly reduced to 15 items	Good (α = 0.92).	22 items version 2 subscales – Intrapersonal α=0,94 – Interpersonal α=0,77) – 15 items version: unidimensional (α=0,933)	NO; the 15-item version was validated in BD	*N =* 335 (15 items version)	Short and unidimensional scale
RAS	41		5 dimensions – Personal confidence and hope (α=0,87) – Willingness to ask for help (α=0,84) – Goal and success orientation (α=0,82) – Reliance to others (α=0,74) – No domination by symptoms (α=0,74)	NO	*N =* 1824	The most widely used PR scale in SMIs
SRS	45		3 dimensions considering PR as a process: Regaining autonomy, Disability management/Taking responsibility, Sense of hope + 3 dimensions considering PR as an outcome: Overall wellbeing, Social functioning/role performance, Helping others	NO	*N =* 471	Developed specifically for chinese-speakers people suffering from SMIs

### Comprehensive Approach to Recovery in BD

#### Through Qualitative Studies

The first articles identified in this review explored the concept of PR in BD in early 2010. Previously, authors were interested in notions that were restricted to the reduction of clinical symptoms. At that time, the term “recovery” was used, referring to clinical recovery and not to the PR process.

One of the main themes of the thematic analysis of the content of qualitative studies was the concept of PR itself and how people living with BD act in relation to PR.

Within this theme, we identified four subthemes:

- First, in 2012, Todd et al. focused on one general question: What does PR mean to people living with BD?
- Three other subthemes emerged from other studies: mental relationships with mood swings, relationships with other people, and finding ways of caring for oneself.

First subtheme: “What does PR mean to people living with BD?”

In 2012, Todd et al. (13) conducted a series of three focus groups with 12 people suffering from BD, asking them what PR meant for them. Four themes emerged: PR is not about being symptom-free but means living a personally fulfilling life alongside one's condition despite mental health symptoms; PR requires taking responsibility for one's own wellness and necessitates moving away from the traditional medical mode; self-management is considered to be one of the key components of recovery; and there is a need to overcome barriers to recovery, including negativity, stigma and taboo. The findings of Todd et al. contributed to the development of the BRQ scale.

Second subtheme: “Mental relationships with mood swings”

The subtheme of mental relations with mood changes highlighted four concepts that were sources of PR (12, 14):

- Acceptance and progressive tolerance of changes in internal states.
- Less extreme control behaviors and less hypervigilance about manic recurrences, contributing to the development of a more coherent and less dependent self-image.
- Good acceptance of the disorder, allowing self-care.
- The need to manage the ambivalence of letting go of manic states.

PR would thus be made up of an equilibrium between acceptance of the disorder and an appropriate relationship with fluctuating thymic states.

Third subtheme: “Relationships with others”

This theme included three concepts: (1) the fundamental need for a social support network that provides support and companionship and (2) helping relationships in which others can alert the person concerning a weakening of mood in (3) a relationship of trust and guidance (14). Moreover, openness to others was established as key to PR: this concept was framed in terms of engendering “trust” and “closeness” with others (12).

Fourth subtheme: “Finding ways of caring for oneself”

Self-care and self-management were highlighted as central features of PR in people with BD.

The main tools of self-care were discussed as follows: accepting that one has a problem to understand it better, gaining experience through both external sources and personal experiences, and having a balanced lifestyle (12).

Veseth et al. insisted on the need to find a fixed point, something to anchor one's life despite constant fluctuations. According to these authors, becoming aware of signals from oneself or others is also a way of self-care: indeed, self-care practices are closely linked to the human and material environment. Furthermore, the authors stressed the importance of agency and responsibility: all participants in their study made “the choice to get better” (14).

**Figure 2:**
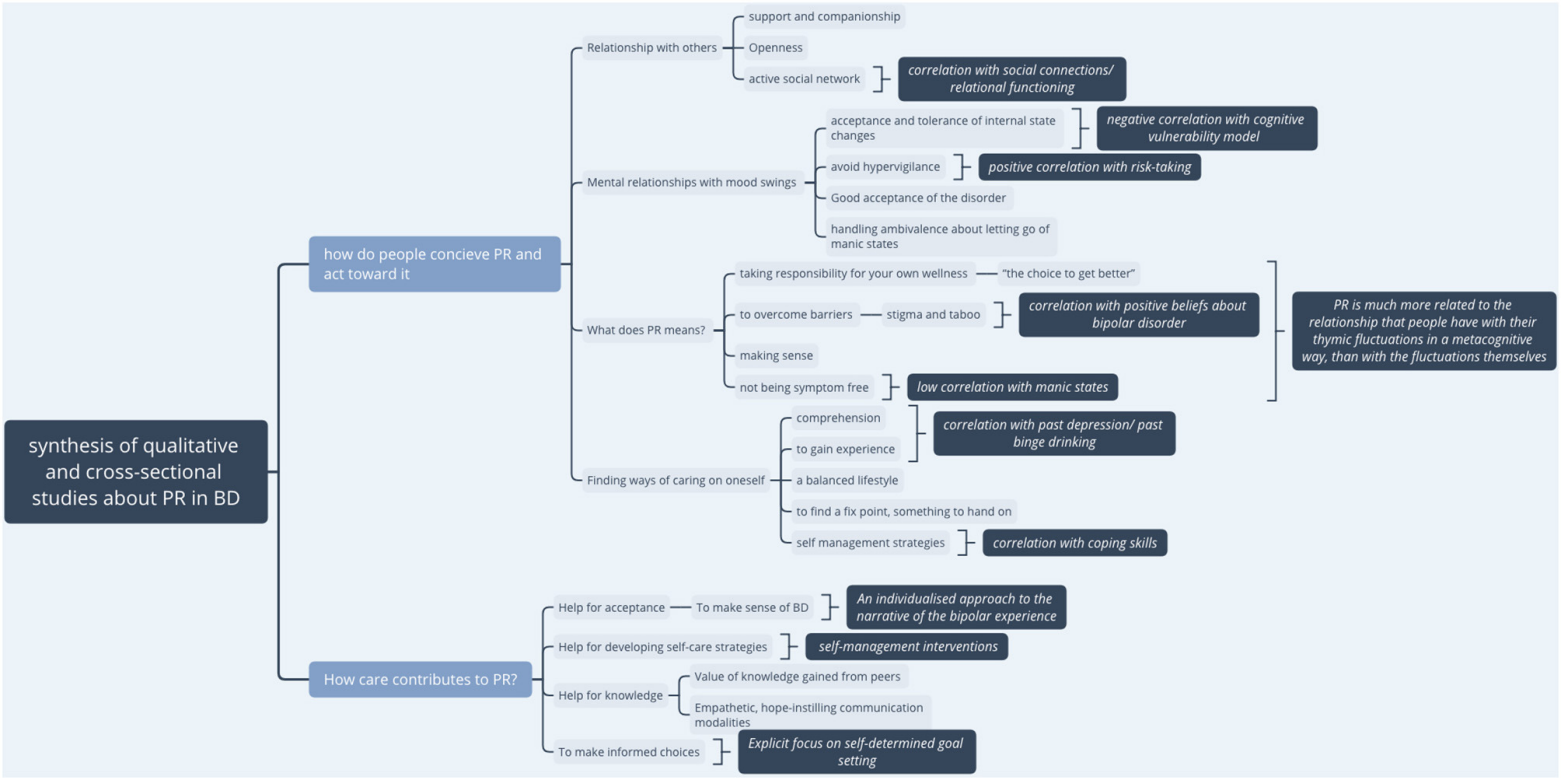
Synthesis of qualitative and cross-sectional studies.

A synthesized presentation of the qualitative results and links with elements of observational studies is presented in Figure 2.

#### Observational Studies Using Psychometric Tools for a Comprehensive Approach to PR

The observational studies used other means to understand the PR process in BD. These studies highlighted three types of factors associated with effective PR in BD by measuring the correlation between scores on PR scales and measures of social, clinical, psychological factors.

##### Social and Professional Factors Associated With PR

Notably, sociodemographic variables such as age and gender did not predict PR in BD (8, 13, 21), but a higher level of education was found to favor PR (26).

Having a meaningful job and having a high level of satisfaction with social roles were the most positively correlated with PR (29). Employment was highly associated with PR (20–22) and predictive of PR at a six-month follow-up (39).

The quality of social support, particularly the frequency of social connections, was also associated with high PR (26). The quality of relational functioning with close relatives, more than the family situation itself, was positively correlated with the level of PR (28).

##### Clinical Factors Correlated With PR in BD

Different studies have shown a significant negative correlation with current depression and anxiety symptoms (8, 21, 22) but a positive correlation with a past experience of depression (8, 22).

There was no correlation with the intensity of manic symptomatology (21) or only a small positive correlation (22, 24, 25).

Another finding was a positive correlation between PR and a young age of diagnosis of BD (19), which was not found by Grover et al. Finally, a history of binge drinking was positively correlated with PR (19).

##### Psychological Factors Associated With PR

Various psychological characteristics were shown to contribute to better PR in people suffering from BD. These characteristics included wellbeing, posttraumatic growth (8), empowerment (19), coping skills including religious values and spirituality (21, 39), and confidence in one's own resources. Low self-stigma, positive beliefs about BD, and good acceptance of mood swings were also associated with PR (22). Additionally, emotion-focused positive ruminations, described as the tendency to respond to positive emotions with recurrent thoughts about positive experiences, were positively correlated with PR (29). Additionally, the level of self-efficacy and self-management skills were related to PR (22).

A higher level of risk-taking was also associated with PR. Risk-taking includes inappropriate but potentially pleasurable activities such as recreational drug use, binge drinking or casual sex (39).

Finally, self-determination was highlighted: the “Respect, Hope, and Self-Directed Empowerment” (RHSE) dimension of the Recovery Elements Assessment Questionnaire (REAQ) was strongly associated with advanced PR. This demonstration reinforced the importance of making informed choices for oneself to facilitate progression to the next stages of PR (19).

In contrast, several factors that inhibit PR were identified. A high level of self-stigma and negative illness models, such as thoughts about uncontrollable mood swings, were correlated with a worse level of PR (21, 22). Higher negative self-dispositional appraisals were associated with lower PR (39). Dampening, which is described as a cognitive strategy of suppression of positive moods to reduce the intensity of positive affect (40), was moderately associated with poor PR (29).

### In What Ways Does Care Contribute to PR?

#### Tools and Modalities of Care Supporting PR in BD

The qualitative studies explored the needs expressed by people suffering from BD to support their PR within the care system.

Three subthemes emerged:

- People expect care to help them accept and find self-care strategies (15). Some experimented with psychotherapies that facilitated self-awareness; it helped to make sense of BD, to understand it better, to develop self-care strategies, and to have a sense of agency (16).
- The value of knowledge gained from peers was highlighted. How knowledge has been shared was more important than the type of knowledge. Empathetic, hope-instilling communication modalities and the embodiment of role models were key (17).

#### Interventional Studies in Which PR Was an Outcome

These studies provided additional data on the levels of PR in psychiatric care.

##### PR as an Outcome

This review included five interventional studies, of which two RCTs targeted PR as a primary outcome.

In all of these studies, PR was measured using the BRQ (8) only.

Other outcomes that were targeted by this clinical research were quality of life, social functioning, thymic symptoms and time to relapse, therapeutic alliance, self-esteem and self-stigma.

##### Type of Therapeutic Interventions Evaluated by These Studies

The different therapeutic interventions studied were web-based self-management interventions (30, 34), a recovery-focused cognitive behavioral therapy (CBT) program (31), a group psychoeducation program (31), and a program combining psychoeducation, CBT and mindfulness (33).

The five published studies that assessed PR as a primary outcome showed significant effectiveness of the interventions for PR. The detailed results of these studies are presented in Table 3.

Jones (31) detailed the particularities of the recovery-focused treatment approach that differ from usual modalities of treatments to support recovery. This therapeutic intervention was developed in partnership with lived bipolar experience.

- An explicit focus on self-determined goal setting rather than relapse prevention (31) and a focus on building a personally meaningful life alongside symptoms (30)
- An individualized approach to the narrative of the bipolar experience, rather than the application of a predetermined model of the bipolar experience to all clients
- Freedom to work within the model provided by the client
- An openness to addressing issues of functioning and comorbidity as well as mood issues
- Work with people to abandon self-critical and/or stigmatizing language.

##### Effects of These Therapeutic Interventions on PR

Jones et al. (31) showed positive effects of their CBT program on PR and on time to relapse, maintained at 15 months follow-up. This impact on relapse was not associated with a change in residual mood symptoms that were not improved by the intervention. The authors postulated that improvement in PR may itself be a possible mechanism for improving relapse: as the BRQ includes self-care and understanding of mood experiences, these may play a role in relapse prevention. The same team (32) showed significant positive effects on PR of a 10-session psychoeducation intervention program “Mood on track”, with a medium effect size (d = 0.52); these results were confirmed by Richardson for a similar 12-session CBT-based psychoeducation program (33).

Self-management tools also seemed to improve PR in people with BD. Todd et al. conducted an RCT showing the benefit of a self-management program, predominantly on PR, with a mean effect size of 0.7 across all outcomes (30). Enrique et al. (34) confirmed these results with a pre-post therapy study with a smaller sample size (*N* = 20) but using a mixed methodology, combining quantitative results and semistructured interviews with patients and carers. The qualitative interviews indicated that patients felt they had improved their awareness and understanding of their illness and felt more empowered.

## Discussion

### General Discussion

This work is, to the best of our knowledge, the first systematic review collecting different types of studies centered on PR in BD, including qualitative works, observational studies, and interventional studies. A review by Murray et al. (41) focused on psychosocial approaches to supporting PR in BD but did not explore the process itself. In addition, Jagfeld et al. recently published a systematic review of only qualitative works centered on PR in BD (6).

This review highlighted several specific characteristics of PR in people suffering from BD. The results of the different studies collected were generally consistent: the studies using psychometric tools confirmed the data of the studies using a qualitative methodology. This observation allows us to reinforce the validity of our results.

### PR in BD: An Emerging Field of Research

We first observed that research on this topic and with this specific population is in its early stages. Our systematic review concerned all research works on this theme published before May 2021 and with no earlier cut-off date. Prior to 2010, in the field of BD research, the term ”recovery" was only used in the literature to refer to “clinical recovery” or “functional recovery”. Research interest in PR in BD has therefore lagged far behind that in the field of schizophrenia.

These observations are in contrast to the growing interest in PR in SMIs, shared by public authorities and mental health users. The COS published by Retzer et al. and selected in this review confirms this interest. A COS is a standardized collection of outcomes recommended to be reported in all controlled trials in a research area for community-based bipolar studies. This COS contained 11 outcomes including PR. PR is thus considered a central outcome for people with BD (18). Interventional research considering PR as a primary outcome in BD is still very limited: only five clinical studies, including two RCTs, were identified. However, five articles that were not selected for this current study presented study protocols on this subject (42–46).

### PR in Relation to Clinical and Functional Recovery

The studies included in our review showed a significant negative correlation with current depression and anxiety, but not with mania. These results are consistent with those of the meta-analysis by Van Eck et al. (1) in schizophrenia, which showed a small to medium association between clinical and personal recovery. Depression would alter the feeling of PR, whereas PR could reduce symptoms and their thymic impact Best, 2020 (47). Among the subdomains of clinical recovery, affective symptoms were the most correlated with PR, and particularly depressive symptoms. Dubreucq et al. (48) longitudinally assessed the overlap and mutual distinctions between clinical and personal recovery, and found that clinical recovery and personal recovery, although distinct constructs, predict each other over time.

Van Eck et al. showed a positive but weak correlation between PR and functional recovery, while Dubreucq et al. put in evidence a mediating effect of quality of life, including various resources such as social support or autonomy. The studies included in our review focusing on BD, showed an important role for social factors, such as a valued role and the quality of social support.

Our results therefore confirm the reciprocal interplay of these different outcomes and the importance of taking them into account in care.

### Specificities of PR in BD in Regard to Other SMIs

Some of the salient features of PR found here are similar to those described in other SMIs [for a review see (7)]: needing to find meaning in life and a fixed point in one's life despite fluctuations (14), taking responsibility for one's health and finding ways to self-manage one's disorders, making “the choice to get better” (12, 13), being employed and having a meaningful role (14, 20, 29, 39). In addition to other SMIs, but counterintuitively concerning mood disorders, PR does not seem to correlate very closely with the symptomatology itself, especially manic states (21, 22). The process seems to be more related to the relationship that people have with their thymic fluctuations than with the fluctuations themselves in a metacognitive way, including resilience (49).

Indeed, our findings provide additional knowledge on more specific elements of PR in BD: first, a new relationship with fluctuating mood states, which includes, on the one hand, an acceptance of this reality and, on the other hand, a decrease in hypervigilance and active struggle against these fluctuations, which may even include risk taking (12, 14, 22, 29, 39). This notion of acceptance had been found by Jagfeld's team in a review of qualitative studies on PR in BD (6). Acceptance concerned a form of ambivalence toward manic symptoms requiring a form of mourning, as well as acceptance of the repeated experience of losses (job, relationships) in connection with extreme mood states. Risk-taking includes inappropriate but potentially pleasurable activities, such as recreational drug use, binge drinking, and casual sex. It has been suggested that low-risk activities that are not associated with high levels of symptoms can be positive experiences and linked to better PR through experimenting and socializing more (39).

Moreover, our review highlights the fact that openness to others, including trust and closeness, seems to be a particularly important element of PR in BD (12, 14, 26). With regard to people with schizophrenia, Jose et al.'s 2015 review about PR in schizophrenia (50) found five themes of personal recovery specific to this disease: the process itself (non-linear, ongoing), self-orientation (e.g., understanding and accepting oneself, returning to a normal state), family relationships, social inclusion and connections, and recovery from illness (no symptoms, good functioning). The recent review by Leendertse (51) showed a strong correlation with the dimensions of hope, empowerment and meaning in life in PR and a lesser correlation with connectedness and identity. It would therefore seem that the notion of connectedness, linked to that of openness to others, is a PR lever that is rather specific to people living with BD compared to those living with schizophrenia. This result suggests that people suffering from BD are less concerned about the autistic symptoms and the social withdrawal present in the negative symptomatology of schizophrenia. Their need for quality social links would therefore be greater to improve their PR.

### The CHIME Model and PR in BD

The CHIME conceptual framework is currently the most widely used framework for describing the PR process (5). However, the data in Leamy's review mainly concerned schizophrenia. The CHIME framework has been criticized for not being sensitive to the specific characteristics of certain populations, especially people with mood disorders, and for omitting the public relations themes of coping and courage or risk taking (for a review, see van Weeghel et al., 2019). While Todd's work in 2012 (13) found dimensions close to those of the CHIME framework, other studies identified new components of PR in this population.

These new elements are of two types. First, the CHIME framework describes elements of PR but does not address the underlying psychological processes. The latter appears to be central in the mechanism of PR in BD, based on the studies in our review. Indeed, the modalities of mental relationships with mood fluctuations have been considered important levers of PR (12). Concordantly, it has been shown (29) that certain regulatory mechanisms of manic or hypomanic states, such as dampening, impede PR. Other authors are exploring cognitive models that may provide explanations for the PR process in cognitive ways. In particular, it seems that a lack of control over fluctuations, acceptance or even a high level of risk-taking may promote PR (39).

Furthermore, the notion of relationships with others is only slightly developed in the CHIME model. However, our results suggest the importance of relationships and quality connections to others (12). Indeed, personal meaning in relationships may be more important in BD recovery than in other SMIs.

Our findings are quite consistent with those of the qualitative literature review conducted by Jagfeld et al. (6). These authors emphasized vocational goals and self-management skills and emphasized the question of relationships with others. Indeed, importance was given to the need to address the tensions in particularly intimate relationships caused by mental health difficulties and to deal with the risk of stigmatization associated with openness. Jagfeld et al. added a “tension” dimension to the CHIME framework, which links the latter subdomain to the issue of the need for acceptance of vulnerability and personal limitations, as well as the need to handle ambivalence about mania. The authors thus proposed a POETIC (Purpose and meaning, Optimism and hope, Empowerment, Tensions, Identity, Connectedness) framework that is supposed to be more specific to PR in BD, according to qualitative data (6).

### Limitations

Our search strategy included three bibliographic databases, which may have limited the number of articles selected. As a result, it is possible that some data of interest could not be collected. We excluded all articles that were not specifically focused on people with BD. In this way, we were able to collect very specific information in a very complex area of research. This choice may also have limited the number of articles selected.

The literature search was limited to peer-reviewed studies available in English or French, which may not represent all the evidence and may have introduced a language bias.

The characteristics of PR in people with BD are in the early stages of exploration. The small number of articles to date may limit the scope of the evidence. Moreover, our work found that there is a lack of RCTs and works with a high level of scientific evidence. It is clear that future work will need to add to current scientific knowledge on the PR process in BD.

### Future Directions and Clinical Implications

Our review has highlighted several possible avenues for future work.

The first avenue concerns the measurement tools of PR in BD. To date, the only scale specifically validated for the context of BD is the BRQ (8), which is the only tool that has been used in clinical research in specific bipolar populations. Our work highlighted that some new components of PR emerged from subsequent qualitative and observational studies. These elements could be taken into account in the construction of other measurement tools complementary to the BRQ.

The second avenue concerns the care tools and “recovery-oriented intervention” in BD. Our findings showed that a number of characteristics of health services supporting resilience, respect, hope and self-directed empowerment supported PR (19).

In 2017, Murray et al. proposed the use of mindfulness tools, such as mindfulness-based cognitive therapy (MBCT) programs or acceptance and commitment therapy (ACT) therapies. These tools would promote acceptance and decrease self-stigma (41). This proposal is consistent with our results; we can indeed hypothesize that these care tools are active in the cognitive relationships maintained with mood swings.

These principles are embodied not only in psychosocial interventions (41) but also in other fields, such as involvement in decision making regarding medication and constrained care. These issues are highly complex and require awareness and vigilance so that the development of self-management does not lead to a decrease in carers' commitment. In contrast, it is a question of finding and developing other, more humanistic, support methods that are a source of recovery. The results of the clinical trials collected in our review were consistent with these data. Recovery-focused care tools were already described (30, 31): they focused on self-determined goals and avoided stigmatizing discourses. This type of care helps people with BD understand and accept their disorders and develop self-care and a sense of agency. We did not find any clinical studies investigating the effects of pharmacological treatments with RA as the primary outcome.

Beyond the care tools themselves, it is also necessary to study communication modalities and the caregiving posture, as well as the partnership with peers in recovery-focused care, that could specifically facilitate PR. In the qualitative study conducted by Tse et al. in 2019 (17), the interviewed patients emphasized the value of learning from peers. Additionally, notions of empathic communication and hope instillation were mentioned. We can link this observation of Tse et al. to the previous results concerning the particular weight of connectedness and openness to others in the PR of people living with BD. Enabling people with BD to develop quality communication skills could be a central goal of care. The goal of recovery-focused care could first be embodied in the caregiving posture itself, through working with peers and valuing experiential knowledge. Additionally, the strengthening of the skills of openness to others, connectedness and bonding with others could be the focus of new care programs, as well as new research perspectives.

## Data Availability Statement

The original contributions presented in the study are included in the article/supplementary material, further inquiries can be directed to the corresponding author.

## Author Contributions

The protocol was designed by MC-E, MB, and LM. The reference screening was performed by MC-E undertaken by MD and BS. Data extraction was performed by MC-E and double-checked by MB and LM. The manuscript was written by MC-E, MB, and LM and reviewed for intellectual content by J-BH and BS. All authors contributed to the article and approved the submitted version.

## Conflict of Interest

The authors declare that the research was conducted in the absence of any commercial or financial relationships that could be construed as a potential conflict of interest.

## Publisher's Note

All claims expressed in this article are solely those of the authors and do not necessarily represent those of their affiliated organizations, or those of the publisher, the editors and the reviewers. Any product that may be evaluated in this article, or claim that may be made by its manufacturer, is not guaranteed or endorsed by the publisher.
